# Presence of pathogenic *Escherichia coli* is correlated with bacterial community diversity and composition on pre-harvest cattle hides

**DOI:** 10.1186/s40168-016-0155-4

**Published:** 2016-03-22

**Authors:** Jessica Chopyk, Ryan M. Moore, Zachary DiSpirito, Zachary R. Stromberg, Gentry L. Lewis, David G. Renter, Natalia Cernicchiaro, Rodney A. Moxley, K. Eric Wommack

**Affiliations:** Delaware Biotechnology Institute, University of Delaware, Delaware Biotechnology Inst., 15 Innovation Way, Newark, DE 19711 USA; College of Veterinary Medicine, Kansas State University, Manhattan, KS USA; School of Veterinary Medicine & Biomedical Sciences, University of Nebraska-Lincoln, Lincoln, NE USA

**Keywords:** STEC, EHEC, O157, Non-O157, 16S rRNA gene, Microbiome, Community profiles, Cattle

## Abstract

**Background:**

Since 1982, specific serotypes of Shiga toxin-producing *Escherichia coli* (STEC) have been recognized as significant foodborne pathogens acquired from contaminated beef and, more recently, other food products. Cattle are the major reservoir hosts of these organisms, and while there have been advancements in food safety practices and industry standards, STEC still remains prevalent within beef cattle operations with cattle hides implicated as major sources of carcass contamination. To investigate whether the composition of hide-specific microbial communities are associated with STEC prevalence, 16S ribosomal RNA (rRNA) bacterial community profiles were obtained from hide and fecal samples collected from a large commercial feedlot over a 3-month period. These community data were examined amidst an extensive collection of prevalence data on a subgroup of STEC that cause illness in humans, referred to as enterohemorrhagic *E. coli* (EHEC). Fecal 16S rRNA gene OTUs (operational taxonomic units) were subtracted from the OTUs found within each hide 16S rRNA amplicon library to identify hide-specific bacterial populations.

**Results:**

Comparative analysis of alpha diversity revealed a significant correlation between low bacterial diversity and samples positive for the presence of *E. coli* O157:H7 and/or the non-O157 groups: O26, O111, O103, O121, O45, and O145. This trend occurred regardless of diversity metric or fecal OTU presence. The number of EHEC serogroups present in the samples had a compounding effect on the inverse relationship between pathogen presence and bacterial diversity. Beta diversity data showed differences in bacterial community composition between samples containing O157 and non-O157 populations, with certain OTUs demonstrating significant changes in relative abundance.

**Conclusions:**

The cumulative prevalence of the targeted EHEC serogroups was correlated with low bacterial community diversity on pre-harvest cattle hides. Understanding the relationship between indigenous hide bacterial communities and populations may provide strategies to limit EHEC in cattle and provide biomarkers for EHEC risk assessment.

**Electronic supplementary material:**

The online version of this article (doi:10.1186/s40168-016-0155-4) contains supplementary material, which is available to authorized users.

## Background

Shiga toxin-producing *Escherichia coli* (STEC) strains are zoonotic pathogens that colonize the lower gastrointestinal tracts of cattle and other ruminants. STEC strains are shed in the feces of these animals, which serve as reservoirs and major sources of foodborne illness [1, 2]. In the USA, foodborne STEC infections are estimated to cause 175,905 illnesses and 21 deaths each year, with 55.2 % of the outbreaks attributable to beef [3, 4]. The STEC serotype O157:H7 (*E.coli* O157) is highly virulent with a 46.2 % hospitalization rate, compared to 12.8 % for that of non-O157 STEC [3]. *E.* coli O157 is the most widely characterized and studied serogroup of STEC [5–7], although 20 to 50 % of infections worldwide are caused by non-O157 serogroups, largely O26, O111, O103, O121, O45, and O145 [8, 9].

Every environment from the cattle gut to the human intestine is an opportunity for these pathogens to encounter and compete with other microbial populations. Commensal indigenous microbes have been shown to mitigate the proliferation of invading pathogens through predation, nutrient competition, and the excretion of antimicrobial compounds [10–12]. In particular, *E. coli* O157 appears to thrive within microbial communities demonstrating lower species diversity [13]. Specifically, in soil and manure environments, microbial diversity is negatively correlated with the invasion of *E. coli* O157 and *Listeria monocytogenes* [12, 14, 15]. These studies suggest that indigenous microbial populations interact, often negatively, with pathogen populations. This apparent protection against invasion is especially evident when the diversity of the microbial community is large enough to occupy a broad spectrum of ecological niches, thereby reducing the likelihood that an alien species could gain traction within the novel environment [11].

In cattle, hides are major contributors to STEC contamination of carcasses, particularly during slaughter [16–19]. As a result, STEC-positive hide samples have been shown to be more predictive of carcass contamination than fecal-positive samples [20, 21]. Interventions focused on the hide, such as dehairing, and washes with water, various chemicals, organic acids, and bacteriophage have been developed to stem STEC contamination [22]. However, the role of the commensal hide bacterial community in preventing or limiting the prevalence of STEC in cattle is unknown.

Enterohemorrhagic *E. coli* or EHEC, a subgroup of STEC, are generally defined as *E. coli* that contain genes that encode Shiga toxin (*stx*) and the locus of enterocyte effacement proteins (e.g., intimin, *eae*) [23]. EHEC within the O157 and six aforementioned non-O157 serogroups cause >90 % of the human STEC infection cases in the USA [24] and have been declared adulterants in raw, non-intact beef by the US Department of Agriculture Food Safety and Inspection Service [25]. To examine whether a connection between EHEC presence and the composition of hide-specific bacterial communities exists, we performed high throughput 16S ribosomal RNA (rRNA) sequencing of bacterial communities from hide samples exhibiting varying degrees of *E. coli* O157 and non-O157 contamination over a 3-month time period.

## Methods

### Sample collection

A total of 576 cattle were sampled over the course of 12 weeks in summer 2013 from a large commercial feedlot operation. Fecal samples were collected at the feedlot, while hide-on carcass surface sponge samples were collected at the abattoir as previously described [26]. Each week, 24 fresh pen-floor fecal samples were collected from each of two cattle pens 12 to 24 h prior to transport to the harvesting plant. At the plant, 24 hide-on carcass samples were collected from cattle in each of the two study pens each week using 11.5 × 23.0-cm sponges (Speci-Sponge®; Nasco, Fort Atkinson, WI) pre-moistened with 35 mL of 0.1 % sterile buffered peptone water (BPW). Sponges were used to sample an area of the hide of 1000 cm^2^, 15 cm from the midline at the level of the diaphragm, after cattle were stunned and bled, prior to hide removal. Sampling procedures as well as characteristics of the study population were described in [26]. Aliquots of 5 g and 5 mL were removed from each fecal and hide sample, respectively, for microbiome analysis. These aliquots were snap-frozen in liquid nitrogen (LN2) within 1 and 2 h of collection of fecal and hide samples, respectively, and stored at −80 °C until completion of a molecular detection assay for EHEC.

### EHEC detection in hide samples

Ninety milliliter of *E. coli* broth (EC; Oxoid Lt., Hampshire, UK) was added to a 35 mL sample-BPW suspension and incubated at 40 °C for 6 h. A previous study [27] tested the prevalence of EHEC in these hide samples using the NeoSEEK™ STEC Detection and Identification test (NS; Neogen Corp., Lansing, MI). The NS utilizes PCR and mass spectrometry to test for >70 independent markers including O-group, Shiga toxin, and intimin. This test determines the presence or absence of EHEC O26, O45, O103, O111, O121, O145, and O157. This work was conducted at GeneSEEK® Inc. (Lincoln, NE). A hide sample positive for EHEC O26, O45, O103, O111, O121, or O145 was grouped into a non-O157 EHEC category, and a sample positive for both non-O157 EHEC and EHEC O157 was grouped into a category called both. A subset (180 hide samples over 8 weeks) of the 576 hide samples that tested positive by NS for EHEC O157, non-O157 EHEC, both, or negative were selected for 16S rRNA bacterial community profiling.

### Microbial community processing

Microbial nucleic acids were extracted from a total of 192 fecal and 180 hide samples collected across the 12-week study via the MO BIO PowerViral DNA/RNA Isolation Kit™. After measuring DNA concentration with the Qubit® Fluorometer, the V3-V4 region of the 16S rRNA gene was amplified using dual-indexed primers [28], with an annealing temperature of 52 °C for 32 cycles. Aliquots of amplicon reactions (3 μL) were electrophoresed on agarose gels to verify amplification. Amplicons were normalized with SequalPrep™ Normalization Plates (Invitrogen Inc., CA, USA), pooled, and then sequenced on the Illumina MiSeq platform (Illumina, San Diego, CA) using the paired-end 250 base pair sequencing protocol. Sequence data discussed in this publication have been uploaded to the DNA Databank of Japan under DDBJ BioProject accession number PRJDB4262.

### Microbial community analysis

#### OTU picking and classification

Raw Illumina sequences from hide and fecal samples were together subjected to quality control and demultiplexing using methods previously described [28]. Processed sequences were classified using open-reference OTU (operational taxonomic units—97 % identity) picking in Qiime [29]. Briefly, the processing steps were as follows: (1) sequences were clustered against GreenGenes v13.8 [30]; (2) sequences that failed to cluster with the reference database were subsequently clustered *de novo*, and the centroids of these clusters were used as reference sequences for the reference database in step 3; (3) sequences that failed the first clustering were clustered against the new reference database generated in step 2; and (4) remaining unclassified sequences were clustered *de novo*. Clustering was performed using UCLUST [31], and resulting sequences were aligned to GreenGenes with PyNAST [29]. OTU classification was reported to the lowest possible taxonomic level.

#### Hide-specific dataset

A dataset containing OTUs specific to hide samples (hereby referred to as Hide Specific OTUs—HSO) was created by first identifying all OTUs that were present in any fecal sample (all fecal OTUs—AFO). These OTUs were then removed from the set of OTUs present in any hide sample, resulting in a set of OTUs that were present in hide samples, but absent from fecal samples (HSO). This hide-specific data set was analyzed in conjunction with the dataset containing all hide OTUs (all hide OTUs—AHO) (Additional file 1: Figure S1).

#### Alpha diversity

Community richness, to assess alpha diversity, was evaluated for each sample using the Chao1 estimator [32] and Faith’s phylogenetic diversity (PD) [33]. Additionally, samples were pooled by various metadata categories (EHEC testing status of hide samples, number of EHEC serogroups, etc.) to examine differences in richness associated with each group. To assess the statistical significance of observed differences in alpha diversity for each metadata group, a jackknifying approach was used. Ten jackknifes were performed for each category, and alpha diversity metrics were calculated for each resulting subset. The level for the jackknifying was set at 95 % of the number of reads of the metadata category with the fewest reads. Significant differences between jackknifed alpha diversity statistics were determined with paired Mann-Whitney tests at an alpha level of 0.05 with Bonferonni correction. These analyses were performed on both the AHO and HSO datasets.

#### Beta diversity

Principal coordinates analysis (PCoA) plots were generated with Emperor [34] using weighted and unweighted UniFrac [35] distance to evaluate large-scale structural shifts associated with each metadata category. Taxonomic profiles for each metadata group were analyzed with Kruskal-Wallis one-way analysis of variance and paired Mann-Whitney tests to identify taxa at significantly higher or lower abundance. OTUs demonstrating significant change were visualized with Cytoscape [36]. Beta diversity analyses were repeated for both AHO and HSO datasets.

## Results

### EHEC identification

For serogroup identification, the NS test indicated that of the 180 hide samples, 41 tested negative for any EHEC, 14 tested positive for *E. coli* O157 only, 84 tested positive for at least one non-O157 serogroup only, and 41 tested positive for *E. coli* O157 and at least one other non-O157 serogroup [27] (Additional file 1: Table S1). Fecal and hide-on carcass samples prevalence at the sample and pen-level and characteristics of the study population were presented elsewhere [26, 27].

### 16S rRNA sequencing

After removing poor quality reads and singleton OTUs, 9,599,163 high-quality 16S rRNA gene sequences were recovered from fecal (7,910,209) and hide (1,688,954) bacterial samples. From these sequences, 97,050 OTUs were classified across the 356 fecal and hide samples at 97 % identity (nine hide samples were removed due to low quality sequencing). Hide samples provided an average of 9877 sequences per sample (Additional file 1: Figure S3), while the mean sequences per sample for fecal samples was 26,963. In ranking the abundance of the OTUs across all hide samples, 80 % of reads were contained in the top 225 clusters, 90 % in the top 554, 95 % in the top 1165, and 99 % in the top 4062 (Additional file 1: Figure S4).

Feces are often found on the hide and likely influenced the occurrence of certain bacterial populations in hide samples. To ensure that trends identified in the dataset containing all hide OTUs (AHO) were robust to these effects, OTUs identified within fecal samples were subtracted from the collection of OTUs found within each hide 16S rRNA amplicon library and designated as hide-specific OTUs (HSO), and all analyses performed on the AHO dataset were repeated on the HSO dataset. After the removal of OTUs also present in fecal samples (all fecal samples—AFO), 2709 hide-specific OTUs (97 % identity) containing 135,202 hide-specific sequences remained (mean 790.655 sequences per sample). Within this collection of sequences, a total of 2709 hide-specific OTUs (97 % identity) were identified across the hide sponge samples. Analysis for the AFO dataset is not within the scope of this manuscript and will be presented elsewhere.

### Bacterial community taxonomy

The principal bacterial families identified in the hide samples (AHO) were *Corynebacteriaceae*, *Ruminococcaceae*, *Lachnospiraceae*, and *Clostridiaceae*, and of these, the *Corynebacteriaceae* was the largest family regardless of testing status (Additional file 1: Figure S5). When the fecal OTUs were subtracted, the dominant bacterial families in the hide-specific dataset (HSO) were *Moraxellaceae*, *Staphylococcaceae*, *Streptococcaceae*, and *Pasteurellaceae*, and of these, the *Staphylococcaceae* was the dominant family in the EHEC positive samples, O157 (39 %), non-O157 (32 %), and both (34 %), while the *Moraxellaceae* was the largest in the EHEC negative sample (33 %) (Additional file 1: Figure S6). *Streptococcaceae* populations comprised a lower relative abundance in *E. coli* O157 (12 %) than in the non-O157 (20 %) and EHEC negative samples (21 %). A comparison of the taxonomic distribution of bacterial species in fecal-specific (FSO), hide-specific (HSO), and shared OTUs is shown in Additional file 1: Figure S2.

### Alpha diversity metrics by testing status

**Fig. 1:**
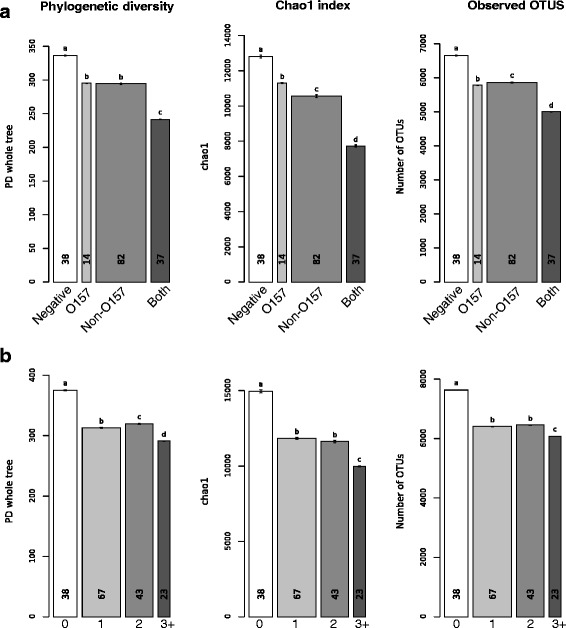
Alpha diversity metrics for samples pooled by EHEC testing status for the all hide OTUs (AHO) dataset. Jackknifed alpha diversity metrics (Faith's phylogenetic diversity, Chao1 estimator, and observed OTUs) were calculated with Qiime’s “alpha_rarefaction.py” script. Faith's phylogenetic diversity, the chao1 index, and observed OTUs were calculated for ten jackknifes at a depth of 95 % of the reads contained in the smallest metadata group. Error bars represent the standard error of the jackknife estimate. The width of the bar shows the number of sequences contained in each metadata category, while the small numbers inside the bars indicate the number of samples in each category. Within each chart, bars with different letters were significantly different at an alpha level of 0.05 with the Bonferroni correction as determined by the Mann-Whitney test. **a** Samples pooled by EHEC testing status into four groups: EHEC negative, *E. coli* O157 positive, non-O157 positive and positive for both *E. coli* O157, and any of the tested non-O157 serogroups. **b** Samples pooled by number or EHEC serogroups present in each sample

In the AHO dataset, alpha diversity was significantly lower (Mann-Whitney test, *P* < 0.05) in cattle hide samples grouped by testing status when any of the EHEC were detected, with the lowest diversity observed in samples that tested positive for both O157 and non-O157 (Fig. 1). The trend continued regardless of whether the diversity was measured by Faith's phylogenetic diversity, the Chao1 species estimator, or observed OTUs (Fig. 1). Additionally, samples were pooled based on number of EHEC serogroups detected, regardless of whether they were O157 or non-O157 positive. When all the OTUs were present, alpha diversity was lower in samples containing one, two, three, or more serogroups as compared to EHEC negative samples (Fig. 1).

The trends seen in the AHO dataset were also seen in the HSO dataset. Alpha diversity was lower for EHEC positive samples (i.e., samples testing positive to either O157, non-O157, or both) when compared to samples without detectable EHEC (Additional file 1: Figure S7), as well as alpha diversity being lower in samples with multiple serogroups as compared to samples without EHEC (Additional file 1: Figure S7).

### Beta diversity metrics by testing status

**Fig. 2:**
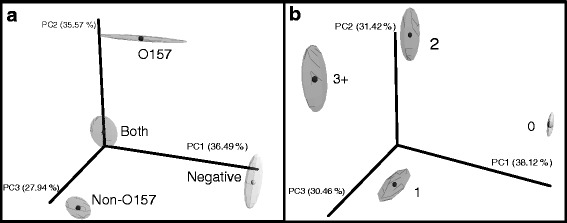
Beta diversity comparison of the bacterial communities by EHEC testing status and number of EHEC serogroups for the all hide OTUs (AHO) dataset. Jackknifed principal coordinate analysis using the unweighted UniFrac metric was performed on all hide sequences with 100 jackknifes of 95 % of the reads of the smallest sample within that metadata category. **a** Samples pooled by EHEC testing status into four groups: EHEC negative, *E. coli* O157 positive, non-O157 positive and positive for both *E. coli* O157, and any of the tested non-O157 serogroups. **b** Samples pooled by number or EHEC serogroups present in each sample. Percentage of variation explained by principal coordinate shown on each of the axis

Beta diversity was compared between testing status (i.e., samples containing either O157, non-O157, or both; Fig. 2a) and the number of serogroups identified within samples (i.e., one, two, three, or more serogroups; Fig. 2b) using a three-dimensional principal coordinate analysis on the AHO and HSO datasets. Regarding the AHO dataset, along the first principal component (PC1), which explained 35 % of the total variability between communities, EHEC positive samples grouped away from EHEC negative samples. Along PC2, which accounted for 36 % of the total variability, samples positive for O157 clustered away from the other categories. When grouped by number of EHEC serogroups identified (Fig. 2b), the negative samples (0 EHEC) and positive samples (one or more EHEC) clustered away from each other along PC1 (38 % of total variability). Samples having one EHEC serogroup population had the same partitioning as O157 along PC1 (31 % of total variability) as samples in the plot grouped by testing status (Fig. 2a). Within the one serogroup group, there were 53 samples (79 %) that contained a non-O157 serogroup compared to 14 samples (21 %) with O157. Thus, the O157 group in Fig. 2a is similar to the one EHEC group in Fig. 2b. Because both groups show similar partitioning, it appeared that the presence of the O157 serotype was driving the separation.

**Fig. 3:**
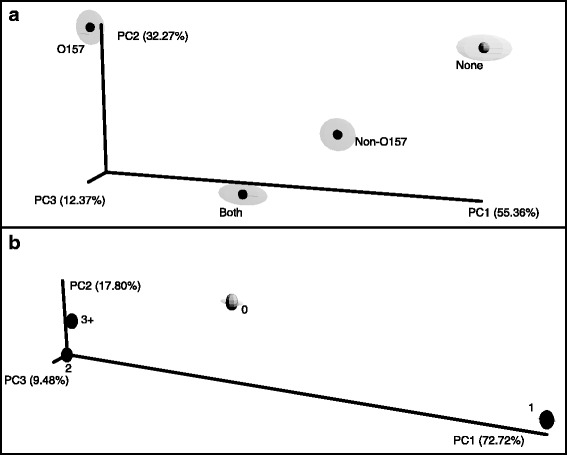
Beta diversity comparison of the bacterial communities by EHEC testing status and number of EHEC serogroups for the hide-specific OTU (HSO) dataset. Jackknifed principal coordinate analysis using the weighted UniFrac metric was performed on all hide sequences with 100 jackknifes of 95 % of the reads of the smallest sample within that metadata category on hide-specific OTUs. **a** EHEC testing status. **b** Number of EHEC serogroups detected in each sample. Percentage of variation explained by principal coordinate shown on each of the axis

When considering the HSO dataset (Fig. 3), there was a lack of distinct separation between the groups (data not shown). As a consequence, the weighted Unifrac measure was employed in the hide-specific analysis (Fig. 3). Along PC1, which explained 55 % of the total variability, EHEC testing status grouped away from each other (Fig. 3a). Similar to Fig. 2b, the samples were grouped by the number of EHEC serogroups detected (Fig. 3b). Along PC1, which explained 73 % of the total variability, the samples with one serogroup and the samples negative for all EHEC (0) clustered away from samples with two or more serogroups.

### OTUs demonstrating significant differences with EHEC testing status

**Fig. 4:**
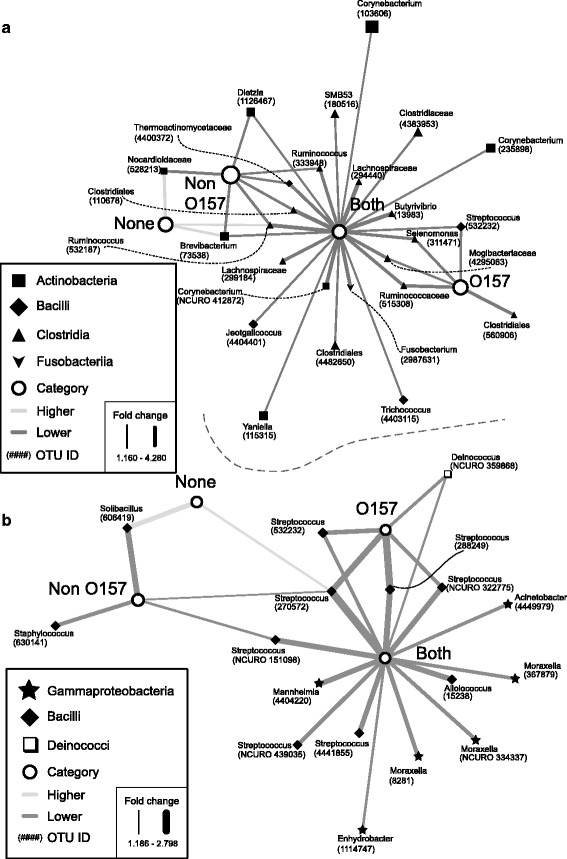
OTUs with significantly different relative abundance according to tesing status. **a** All hide OTUs (AHO). **b** Hide-specific OTU (HSO). *White circles* indicate the testing status of groups of samples: EHEC negative; O157 only; non-O157 only; and both non-O157 and O157. *Black nodes* are OTUs with significantly higher (*P* < 0.05) or lower relative abundance. The shape of OTU nodes is based on class: *black diamond Bacilli*, *black up-pointing triangle Clostridia*, *black square Actinobacteria*, *black star Gammaproteobacteria*, *white square Deinococci*, *black down-pointing triangle Fusobacteria*. Labels indicate the genus of the OTU. OTU numbers from the cluster analysis are indicated in parentheses. A *dark gray line* represents lower average relative abundance. *Light gray lines* indicate higher relative abundance. Edge width is proportional to fold-change, with a *thicker line* representing a higher shift in abundance. When genus information was not available, family or order is indicated

In our study, we use the typical convention of defining OTUs as clusters of reads at 97 % similarity. Each OTU that follows is labeled with the most granular taxonomic classification available. OTUs occurring at significantly higher or lower relative abundance (*P* < 0.05) depending on testing status were identified in both the AHO and HSO datasets (Fig. 4). No OTUs were identified as occurring at a higher relative abundance in EHEC-positive samples in either dataset (Fig. 4), due to the decrease in diversity as depicted in Fig. 1. In the AHO dataset, the “both” group of samples had 23 OTUs at a relative lower abundance. Of these, four were shared with “O157” positive group, *Streptococcus* OTU #532232, *Selenomonas* OTU #311471, *Mogibacteriaceae* OTU #4295063, and *Rumiococcaceae* OTU #515308, and six were shared with “non-O157” positive group, *Dietzia* OTU #1126467, *Ruminococcus* OTU #333948 and #532187, *Thermoactinomycetaceae* OTU #4400372, *Clostridales* OTU #110678 and *Brevibacterium* OTU #73538. The remaining 13 were not shared with any other groups of samples that were categorized by EHEC prevalence: *Lachnospiraceae* OTU #299184 and # 294440, *Jeotgalicoccus* OTU #4404401, *Fusobacterium* OTU #2987631, *Trichococcus* OTU #4403115, *Clostridiales* OTU #4482650, *Corynebacteria* OTU #412872, #235898, and #103606, *Yaniella* OTU #115315, *Clostridiaceae* OTU #4383953, *Butyrivibrio* OTU #13983, and SMB53 OTU #180516. All the OTUs at lower or higher relative abundance in the EHEC negative (“none”) and “non-O157” groups were shared with another group. However, there was one OTU at lower abundance in the “O157” positive group that was not shared with any of the other EHEC metadata categories, *Clostridales* OTU #560906. The EHEC negative group, “none,” had three OTUs that were at greater relative abundance, which were all at lower relative abundance in “non-O157” positive samples, *Brevibacterium* OTU #73538, *Ruminococcus* OTU #532187, and *Nocardioidaceae* OTU #528213.

The hide-specific dataset (HSO) had fewer OTUs showing significant (*P* < 0.05) differences in relative abundance with EHEC testing status. This was due to the reduction in the number of samples and OTUs in the HSO dataset (Fig. 4b). The groups were similarly distributed as in Fig. 4a, with the EHEC-positive categories (“both,” “O157,” and “non-O157”) having only OTUs at lower relative abundance and the EHEC negative group “none” having only OTUs at higher relative abundance. The “both” group, as in the dataset containing fecal samples, had the largest abundance of altered OTUs at 15. Five of these were shared with the “O157” and two with “non-O157” groups. Almost all of these OTUs were part of the genus *Streptococcus* (“O157” OTUs #270572, #288249, #322775, and #532232; “non-O157” OTUs #270572 and #151098), with the exception of *Deinococcus* OTU #359868 shared with “O157.” One of the *Streptococcous* OTUs (#270572) was also at a higher relative abundance in the “none” group. The remaining nine OTUs that were at lower relative abundance in the “both” group were as follows: *Mannheimia* OTU #440422, *Enhydrobacter* OTU #1114747, *Moraxella* OTU #8281, #334337, #367879, *Alloiococcus* OTU #15238, *Acinetobacter* OTU #4449979, and two more *Streptococcous* OTUs (#4441855 and #439035). There was also one “non-O157” OTU that was at lower relative abundance, *Staphylococcus* OTU #630141, and not shared with any other group. Only one other OTU demonstrated higher relative abundance in the hide-specific “none” group, *Solibacillus* OTU #606419, and was also at relative lower abundance in the “non-O157” group.

## Discussion and conclusions

Current research suggests that the indigenous microbial community of the skin is on the forefront of defense against the growth of invading bacteria [37, 38]. For instance, *Staphylococcus epidermidis*, a commensal bacterium found on the skin of humans*,* has the ability to prevent the colonization of pathogens, such as *S. aureus* and group A *Streptococcus,* through the binding of keratinocyte receptors and the production of phenol-soluble modulins and other antimicrobial peptides [39–42]. Additionally, increased diversity on healthy intact skin in humans has been speculated to reduce the spread of opportunistic pathogens present in wounds [43]. This complex and varied ecosystem is poorly understood in cattle and may provide a rich source of information for the mitigation of pathogen spread and colonization.

Our analyses showcased the relationship between the composition of hide bacterial communities and the prevalence of EHEC contamination on cattle hides. To test whether these correlations seen in the ASO dataset were truly associated with hide bacterial populations and were not a result of fecal bacterial contamination, OTUs identified in cattle fecal bacterial communities sampled from the same groups of cattle were removed to create a hide-specific bacterial community (HSO). Bacterial families known to inhabit skin and soil environments dominated the HSO group (Additional file 1: Figure S6), while the original dataset (AHO) was dominated by families found in the digestive tract of mammals (Additional file 1: Figure S5). Alpha diversity metrics indicated an association between low bacterial diversity and EHEC occurrence on cattle hide (Fig. 1, Additional file 1: Figure S7). Because our data is observational, we can only hypothesize about the cause of this low diversity trend with EHEC contamination. For instance, it is difficult to assess whether a genomic adaption within the pathogen enabled it to outcompete autochthonous hide microbiota or whether a disturbance presented EHEC populations with the opportunity to colonize an unoccupied ecological niche within the hide community.

Strains of STEC are pathogens that carry a number of genes that enable them to survive within the low pH conditions of the stomach and within nutrient-limited environments [44]. Almost 20 % of the *E. coli* O157 genome is composed of foreign DNA not present in commensal *E. coli* K-12 genome [45]. This additional reservoir of genomic DNA may provide the pathogen with the ability to survive and compete in novel environments, like cattle hides. However, a healthy commensal microbiome may overcome the genomic plasticity of STEC. Generally, antagonism between bacteria can occur indirectly or directly, via mechanisms such as competitive exclusion, the production of antimicrobial agents, or the modification of the environment to unfavorable conditions [46].

It is thought that in an established microbial community, many if not all of the available environmental niches are filled, generating a protective barrier against colonization by an invading pathogen [11]. Therefore, a diverse bacterial community may prevent EHEC from colonizing in a novel environment. Researchers have found that competitive interaction with enteric bacteria can displace or inhibit the growth of *E. coli* O157 in vitro [47–49]. For instance, one study reported the survival of *E. coli* O157 decreased by 20- to 30-fold in the presence of *Enterobacter asburiae*, a competitor for the carbon and nitrogen substrates used by the pathogen [50]. The reduced diversity that was present in a subset of our hide samples may have enabled the pathogen to gain a foothold in a vacant niche (Fig. 1). Alternatively, commensal hide bacterial species may have inhibited the invasion of EHEC populations more directly through the production of antimicrobial compounds such as organic acids and bacteriocins.

In the AHO dataset, the bacterial OTUs that were at higher relative abundance in the absence of EHEC were *Ruminococcus*, *Brevibacterium*, and *Nocardioidaceae*, while in the HSO dataset, the bacteria at higher relative abundance were *Soilbacillus* and *Streptococcus* (Fig. 4). Looking at the types of compounds produced by these bacterial taxa, we can build hypotheses about the possible impact these groups may have had on STEC populations. Several *Ruminococcus* spp. (e.g., *Ruminococcus albus*, *Ruminococcus flavefaciens*) can produce fermentation products such as succinate acetate, formate, and ethanol in the gut, some of which have been shown to impact the survival of STEC [51]. *Ruminococcus,* along with *Clostridium* and *Bacteroides*, have also been identified as members of the human microbiota that can inhibit *stx*2 production and, thus, potentially limit STEC (and EHEC) propagation [52].

Interestingly, a species of *Soilbacillus*, *Solibacillus silvestris,* has the ability to degrade *N*-acylhomoserine lactones (AHLs) [53]. AHLs are widely used for the cellular communication phenomena known as quorum sensing in Gram-negative bacteria. *E. coli* do not produce AHLs themselves, but utilize the AHL receptor of the LuxR family, SdiA. SidA receptors recognize AHLs produced from surrounding bacteria to assess the environment and modulate gene expression as necessary [54]. Previous research has demonstrated that AHL perception in *E. coli* O157 is utilized for intestinal colonization in cattle and is speculated to be critical in *E. coli* survival outside the host [55, 56]. While the role of AHLs in hide colonization is unknown, *S. silvestris* and other AHL-degrading bacterial populations may be interfering with *E. coli* O157 ability to perceive commensal bacteria, thereby decreasing their chances of a successful invasion.

The other group of OTUs identified as having higher prevalence in EHEC negative samples and lower prevalence in O157 positive samples were OTUs belonging to the genus *Streptococcus.* In fact, within the hide-specific dataset, (HSO) seven different *Streptococcus* OTUs were recognized as having lower relative abundance in samples containing EHEC serogroups (Fig. 4). Certain species of *Streptococcus* (e.g., *Streptococcus thermophilus*, *Streptococcus bovi*, etc.*)* produce lactic acid. In addition to lowering the pH, lactic acid has been shown to cause permeabilization of the outer membrane of Gram-negative bacteria, including *E. coli* O157 [57]. Lactic acid-producing bacteria (LAB) have been widely studied as probiotics to inhibit *E. coli* O157, with the majority of these experiments involving *Lactobacillus* spp. [58, 59]. High levels of lactic acid bacteria were shown to inhibit the growth of *E. coli* O157 in ground beef [60] and experimentally infected cattle saw a decrease in *E. coli* O157 shedding when given a probiotic culture of *Streptococcus bovis* and *Lactobacillus gallinarum* [61]. Additionally, sheep fed a cocktail of probiotic bacteria including several *Lactobacillus spp.*, *Streptococcus thermophilus* and *Enterococcus faecium* showed a reduction in non-O157 STEC fecal shedding [62]. Thus, *Streptococcus* may be a useful bacterium in the prevention of STEC colonization in the gut and on the hide.

Despite being a minor community member on the hide and in feces, EHEC populations appear to correspond with drastically different community compositions. This can result in differences between communities containing different EHEC serogroup populations (e.g., O157 versus non-O157) (Figs. 2 and 3). These data suggest that it may be advisable to incorporate bacterial community profiling data into quantitative models of EHEC risks. Going a step further, future investigations should examine the possible use of individual bacterial strains or mixtures of strains as biotherapeutic agents to mitigate EHEC contamination. Through a more complete mechanistic understanding of how commensal microbes on cattle hides prevent EHEC colonization, we may be able to lessen the food safety risks in beef processing associated with these dangerous human pathogens.

## Additional file

Additional file 1:
**Supplementary information.** (PDF 6379 kb)
