# Supplementary material for: Presence of pathogenic Escherichia coli is correlated with bacterial community diversity and composition on pre-harvest cattle hides
Source: Microbiome. 2016 Mar 22;4:9. doi: 10.1186/s40168-016-0155-4 (PMC4802634; doi:10.1186/s40168-016-0155-4)
Supplement: Additional file 1: — Supplementary information. (PDF 6379 kb) [file 40168_2016_155_MOESM1_ESM.pdf]

Supplementary Figures and Tables

Presence of pathogenic *Escherichia coli* is correlated with bacterial community diversity and composition on pre-harvest cattle hides

Jessica Chopyk[1], Ryan Moore[1], Zachary DiSpirito[1], Zachary R. Stromberg[1], Gentry L. Lewis[1], David G. Renter[2], Natalia Cernicchiaro[2], Rodney A. Moxley[3], and K. Eric Wommack[1*]

[1]Delaware Biotechnology Institute, University of Delaware, Newark, Delaware,
[2]College of Veterinary Medicine, Kansas State University, Manhattan, Kansas,
[3]School of Veterinary Medicine & Biomedical Sciences, University of Nebraska-Lincoln, Lincoln, Nebraska

Running title: Cattle hide bacterial diversity and *E. coli*

Corresponding Author's information

*To whom correspondence should be addressed.

Address: Delaware Biotechnology Inst., 15 Innovation Way, Newark, Delaware 19711

(Tel): (302) 831-4362

(Fax): (302) 831-3447

(E-mail): wommack@dbi.udel.edu

Supplementary figures

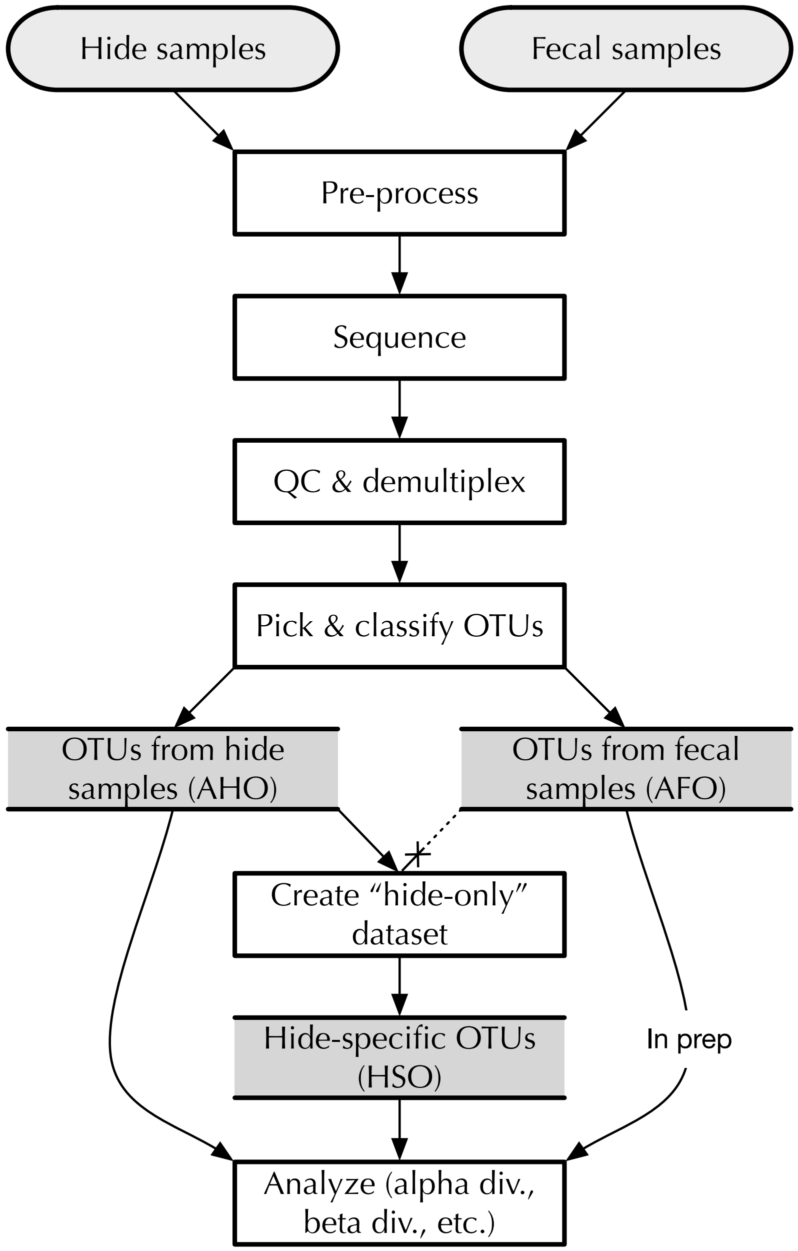

Fig. S1: Workflow for creating the hide-specific OTU dataset. Hide and fecal samples were pre-processed, sequenced, and post-processed as described in the Methods & Materials section. After OTU picking and classification the OTUs from the all fecal OTU dataset (AFO) were subtracted from the all hide OTU dataset (AHO) to produce the hide-specific OTU dataset (HSO).

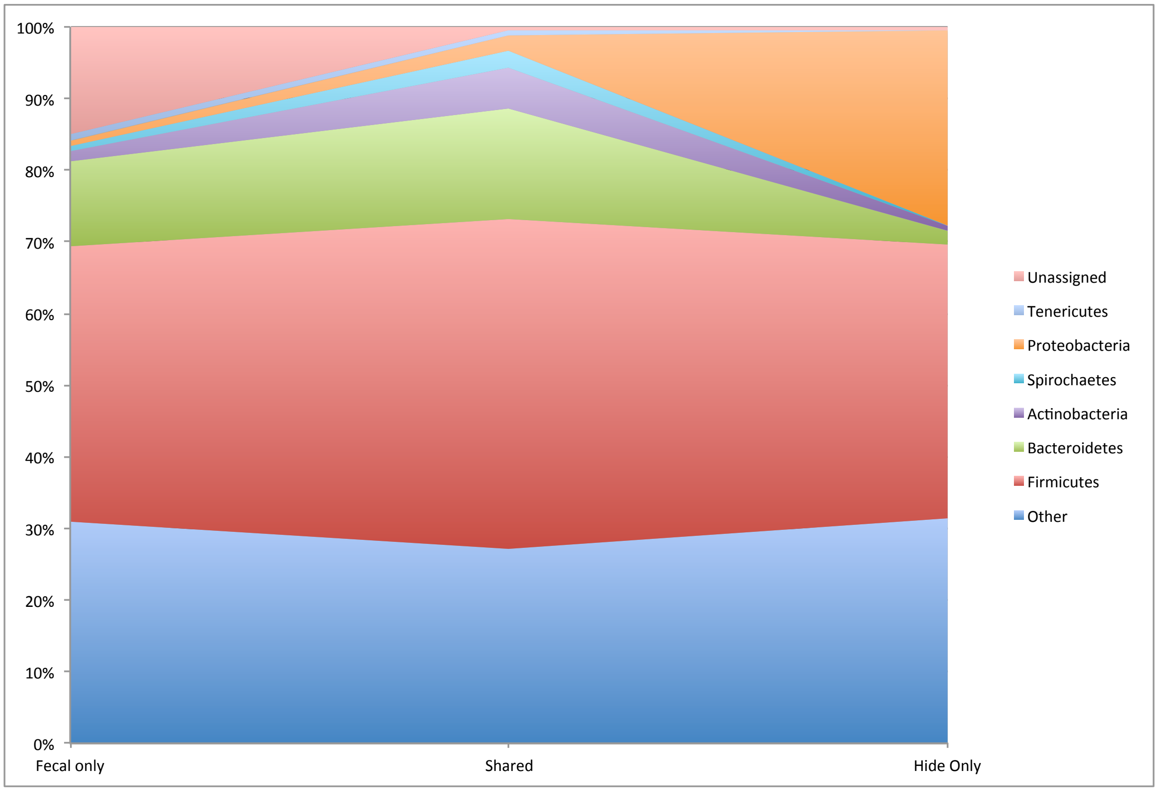

Fig. S2: Comparison of taxonomic distribution of bacterial species in fecal-specific, hide-specific, and shared OTUs. Relative abundance of OTUs specific to fecal samples, hide samples and OTUs shared between fecal and hide samples is shown. Only phyla that were present at a relative abundance greater than or equal to 1% in any of the three categories are named, with the remaining phyla grouped into "Other".

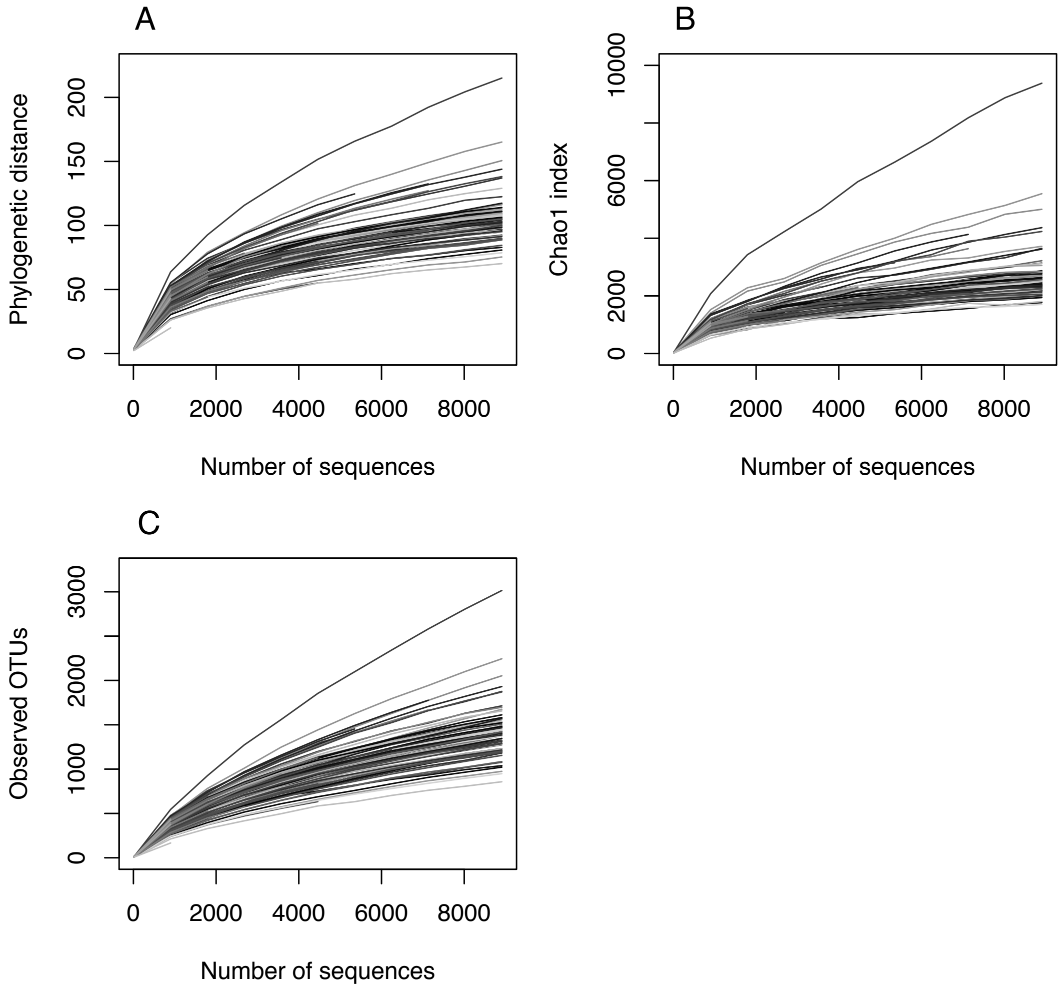

Fig. S3: Hide sample rarefaction curves. Rarefaction curves for 171 hide samples using Faith's phylogenetic distance (A), the Chao1 index (B), and raw OTU counts (C). Each line represents a separate hide sample, with colors added to aid in visually distinguishing samples.

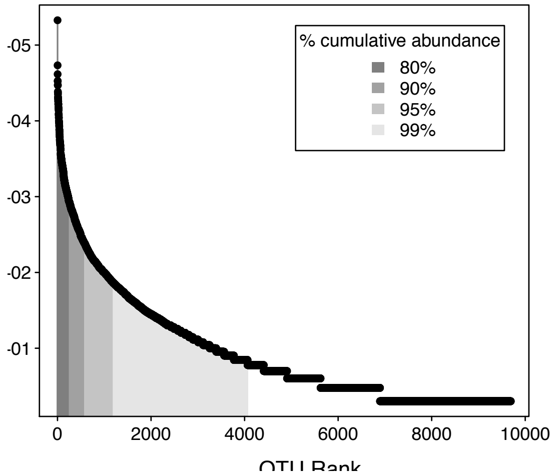

Fig. S4: Rank abundance of OTUs. Rank for OTUs occurring at least twice. The shaded regions under the curve represent the percent abundance contained in that section of the curve. Eighty percent, 90%, 95%, and 99% of the abundance was contained in the top 225, 554, 1165, and 4062 OTUs, respectively.

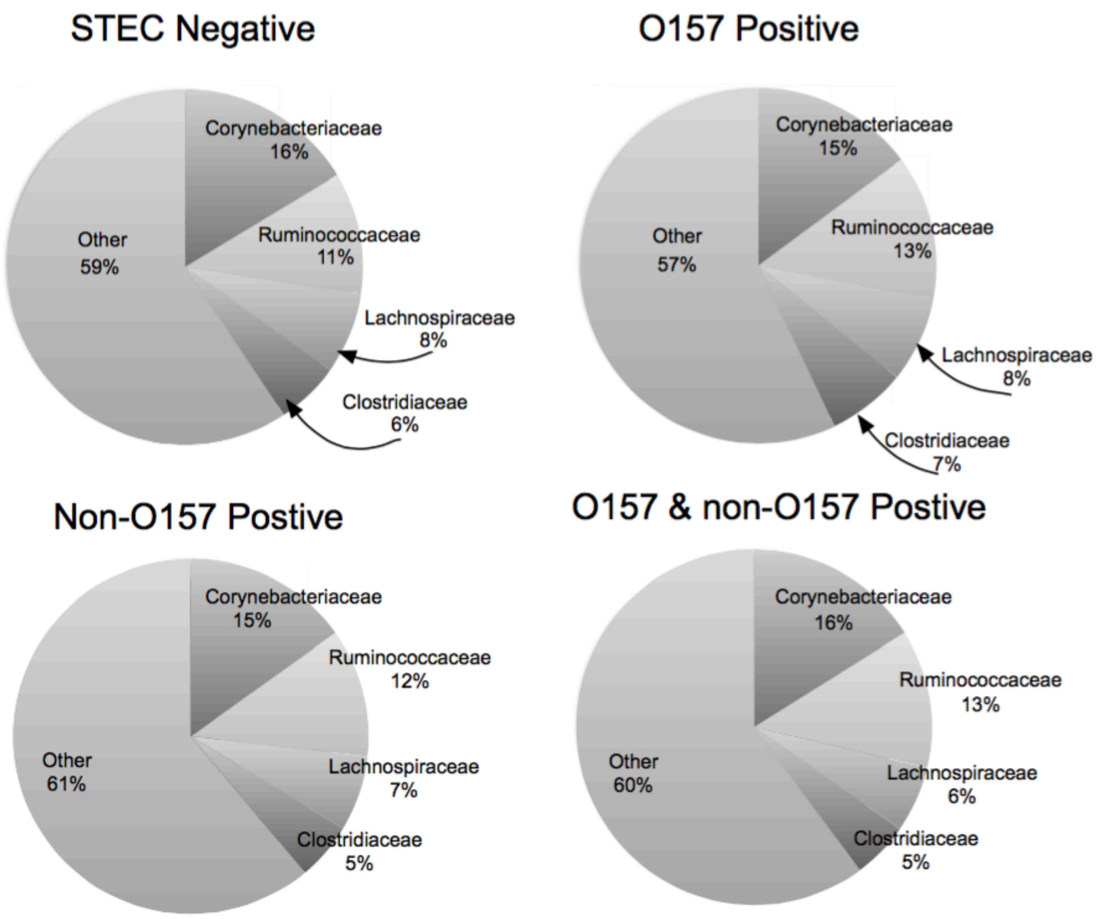

Fig. S5: Taxonomic distribution of bacterial families from hide samples according to testing status for the all hide OTU dataset (AHO)**.** Relative abundance charts were based on distributions of bacterial families as a percentage of the total number of classified 16S rRNA gene sequences. Samples were pooled based on EHEC testing status (EHEC negative, O157 positive, non-O157 positive, and both).

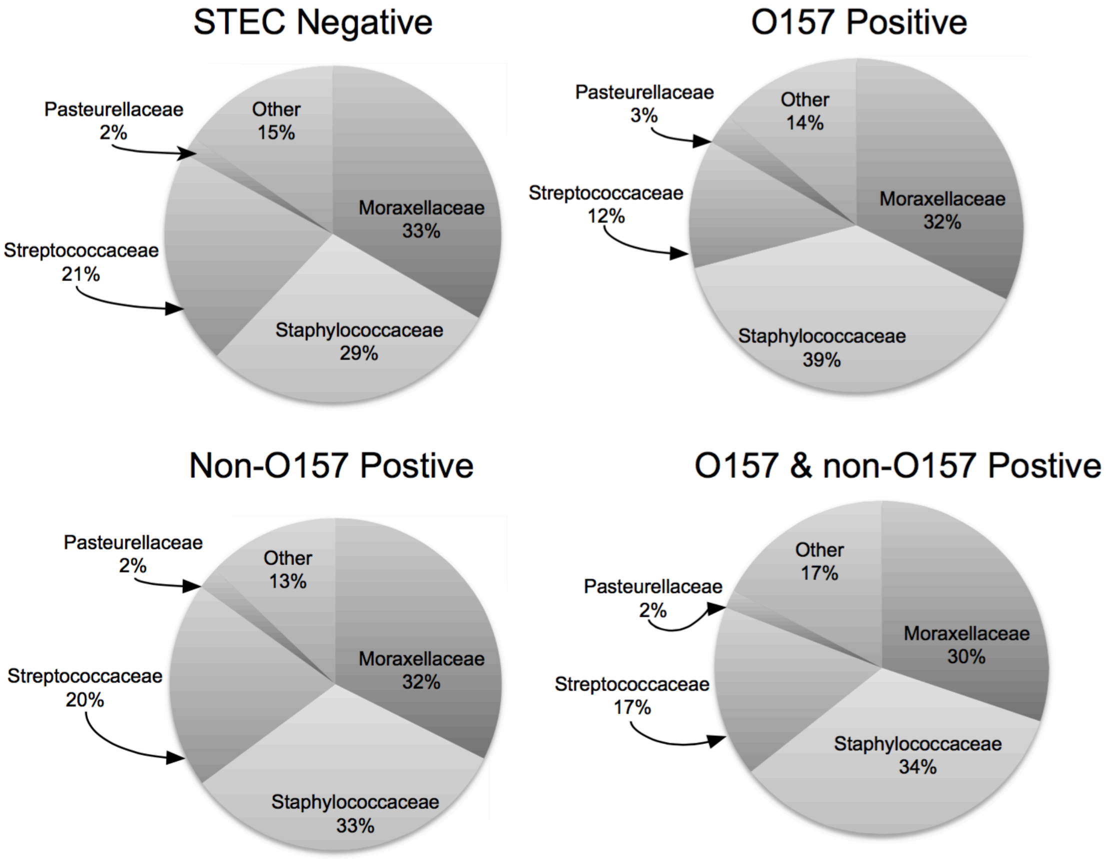

Fig. S6: Taxonomic distribution of bacterial families from hide samples according to testing status for the hide-specific OTU dataset (HSO)**.** Relative abundance charts were based on distributions of bacterial families as a percentage of the total number of classified 16S rRNA gene sequences. Samples were pooled based on EHEC testing status (EHEC negative, O157 positive, non-O157 positive, and both).

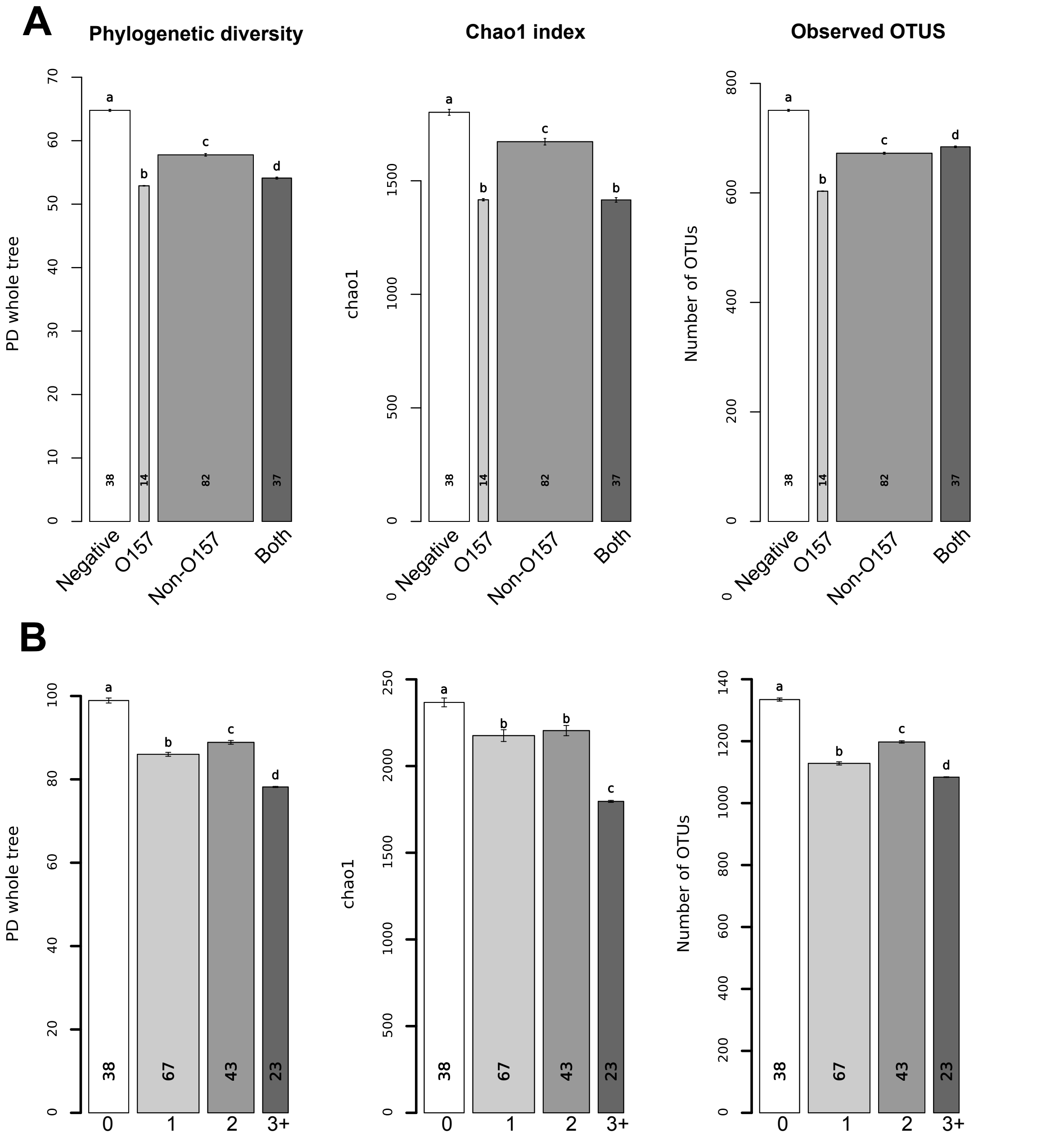

Fig. S7: Alpha diversity metrics for samples pooled by testing status for the hide-specific OTU dataset (HSO)**.** Alpha diversity tested after fecal OTUs were removed. Panel A), samples were pooled by EHEC testing status into four groups: EHEC negative, *E. coli* O157 positive, non-O157 positive and positive for both *E. coli* O157 and any of the tested non-O157 serogroups. Panel B) samples were pooled by number or EHEC serogroups present in the sample.
